# Mutant p53 promotes ovarian cancer cell adhesion to mesothelial cells via integrin β4 and Akt signals

**DOI:** 10.1038/srep12642

**Published:** 2015-07-30

**Authors:** Jong-Gyu Lee, Ji-Hye Ahn, Tae Jin Kim, Jae Ho Lee, Jung-Hye Choi

**Affiliations:** 1 Department of Life & Nanopharamceutical Science, Kyung Hee University, Seoul 130-701, South Korea; 2 Department of Oriental Pharmacy, College of Pharmacy, Kyung Hee University, Seoul 130-701, South Korea; 3 Department of Obstetrics and Gynecology, Cheil General Hospital and Women’s Healthcare Center, Dankook University College of Medicine, Seoul 100-380, South Korea; 4 Laboratory of Molecular Oncology, Cheil General Hospital and Women’s Healthcare Center, Dankook University College of Medicine, Seoul 100-380, South Korea

## Abstract

Missense mutations in the *TP53* gene resulting in the accumulation of mutant proteins are extremely common in advanced ovarian cancer, which is characterised by peritoneal metastasis. Attachment of cancer cells to the peritoneal mesothelium is regarded as an initial, key step for the metastatic spread of ovarian cancer. In the present study, we investigated the possible role of a p53 mutant in the mesothelial adhesion of ovarian cancer cells. We found that OVCAR-3 cells with the R248 *TP53* mutation (p53^R248^) were more adhesive to mesothelial Met5A cells than were A2780 cells expressing wild-type p53. In addition, ectopic expression of p53^R248^ in p53-null SKOV-3 cells significantly increased adhesion to Met5A cells. Knockdown of mutant p53 significantly compromised p53^R248^-induced cell adhesion to Met5A cells. Microarray analysis revealed that several adhesion-related genes, including integrin β4, were markedly up-regulated, and certain signalling pathways, including PI3K/Akt, were activated in p53^R248^ transfectants of SKOV-3 cells. Inhibition of integrin β4 and Akt signalling using blocking antibody and the inhibitor LY294002, respectively, significantly attenuated p53^R248^-mediated ovarian cancer-mesothelial adhesion. These data suggest that the p53^R248^ mutant endows ovarian cancer cells with increased adhesiveness and that integrin β4 and Akt signalling are associated with the mutation-enhanced ovarian cancer-mesothelial cell adhesion.

The *TP53* gene, encoding the p53 tumour suppressor, is the most frequent target for mutation in human cancer^1^. Most cancer-associated *TP53* mutations are missense mutations that result in overexpression of the full-length p53 protein with only a single amino acid substitution. In addition to the loss of normal p53 function through deletion or intragenic mutation, a class of ‘gain-of-function’ mutants exists^2^, in which the encoded proteins are endowed with oncogenic properties that actively drive tumour progression^3^. Indeed, emerging evidence suggests that mutant p53 is involved in genomic instability, aberrant cell cycling, invasion, metastasis, and drug resistance^4^. Thus, p53 mutations have been identified as potential prognostic/predictive markers and targets for therapeutics^5^.

Ovarian cancer is the most lethal gynaecological malignancy in developed countries. Ovarian cancer affects approximately 204,000 women per year worldwide and is responsible for approximately 125,000 deaths^6^. The majority of women with ovarian cancer are diagnosed at a late stage when the cancer has spread beyond the confines of the ovary. Thus, most deaths from the disease are due to metastases that are resistant to conventional therapies. Metastatic spread of ovarian cancer is characterised by ascites and widespread peritoneal implantation. The initial, key step of ovarian cancer metastasis seems to be the attachment of ovarian cancer cells to the layer of mesothelial cells that cover the peritoneal cavity. However, the molecular mechanisms of ovarian cancer-mesothelial adhesion are poorly understood.

Olivier *et al*. have reported that somatic mutations in the *TP53* gene are the most frequent (47.8%) in ovarian cancer among all other sporadic cancers^7^. In fact, alterations of the *TP53* gene are the most common genetic events in advanced ovarian cancer. According to the p53 data base (http://www-p53.iarc.fr/) of the International Agency for Research on Cancer (IARC), most *TP53* mutations in ovarian cancer are, like those in other cancers, missense mutations (>87.56%), which mainly cluster in the DNA binding domain with hotspots at codons 175, 248, and 273. Despite the prevalence of *TP53* mutations in ovarian cancer and the accumulating evidence for gain-of-function cancer-associated *TP53* mutations, little is known about the role of p53 mutants in ovarian cancer development and progression. In addition, to the best of our knowledge, there is no report of an investigation of an involvement of p53 mutants in peritoneal mesothelial adhesion, a key step for the metastatic spread of several cancers, including ovarian and colorectal cancer. In this study, we investigated whether a p53 hotspot mutant, p53^R248^, plays a role in the mesothelial adhesion of ovarian cancer.

## Results

### Mutant ovarian cancer cells expressing p53^R248^ showed an increased adhesion to mesothelial cells

**Figure 1:**
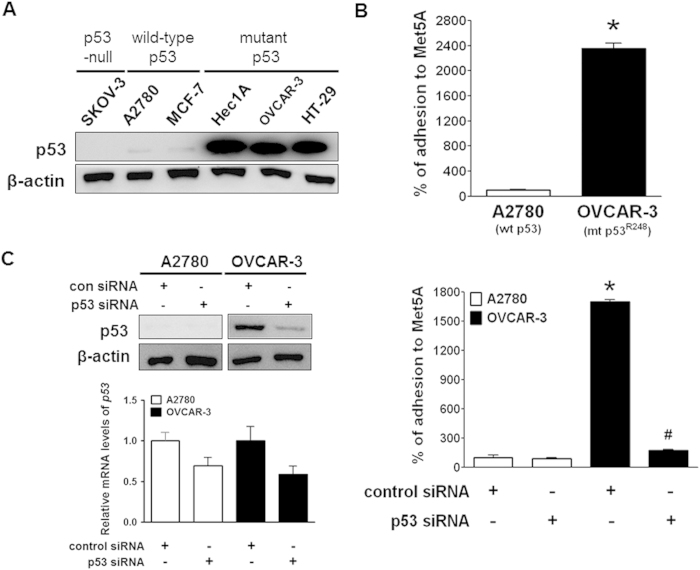
Adhesion of A2780 and OVCAR-3 ovarian cancer cells to mesothelial Met5A cells. (**A**) Western blot assays were performed to measure the p53 protein levels in various cancer cells, including ovarian cancer cells (SKOV-3, A2780, and OVCAR-3 cells); MCF-7, breast cancer cells; Hec1A, endometrial cancer cells; and HT-29, colon cancer cells. (**B**) Attachment assays were performed to investigate the adhesive ability of A2780 and OVCAR-3 cells after probing with CellTracker^TM^. After 60 min of incubation at 37 °C with a Met5A cell layer, the total fluorescence in each well was imaged by fluorescence micro-photography. *P < 0.05 compared with the A2780 group. (**C**) A2780 and OVCAR-3 cells were transiently transfected with p53 siRNA (100 nM) or control siRNA (100 nM), followed by an attachment assay. *P < 0.05 compared with the control siRNA-transfected A2780 group and ^#^P < 0.05 compared with control siRNA-transfected OVCAR-3 group.

The vast majority of cancer-associated p53 mutants are full-length proteins, typically with only a single amino acid substitution, which tend to accumulate in the tumour cells and reach steady-state levels that greatly exceed those of wild-type p53 (wt p53) in noncancerous cells^8^. We measured the p53 protein levels in cancer cell lines with various p53 characteristics: p53-null (SKOV-3), wild-type p53 (A2780 and MCF-7), and mutant p53 (Hec1A, OVCAR-3, and HT-29)^1^,^9^ (Fig. 1A). As previously reported, mutant p53 protein was expressed in excess in Hec1A, OVCAR-3, and HT-29 cells compared with its wild-type counterpart in A2780 and MCF-7 cells, suggesting that the p53 mutants may play active roles in the tumour cells rather than just being relics of wild-type p53 inactivation.

It has been reported that ovarian cancer cells interact with peritoneal mesothelial cells as the disease progresses^10^,^11^. Despite accumulating evidence for p53 gain-of-function mutants participating in various aspects of cancer development and progression, their roles in cancer adhesion are poorly understood. Thus, we investigated whether a p53 mutant with a change at codon 248 (denoted p53^R248^), which is one of the hot spots for ovarian cancer-associated p53 missense mutations, plays a role in ovarian cancer adhesion to the mesothelium, a key step in the malignant progression of the disease. The A2780 cell line expressing wild-type p53 and the OVCAR-3 cell line expressing mutant p53^R248^ are both epithelial ovarian cancer cell lines^12^,^13^. Adhesion of OVCAR-3 cells to human mesothelial Met5A cells was approximately 24-fold higher than that of A2780 cells (Fig. 1B). This observation indicated that the ovarian cancer cells harbouring mutant p53^R248^ may have greater adhesive activity than those harbouring wild-type p53. To determine whether the increased levels of the mutant p53 are involved in cell adhesion, we measured cell adhesion after the knockdown of the mutant p53 using p53 siRNA in OVCAR-3 and A2780 cells. We found that a significant decrease in the adhesion occurred only in OVCAR-3 cells but not in A2780 cells (Fig. 1C). These data suggested that mutant p53^R248^ may be involved in mesothelial adhesion of ovarian cancer cells.

### The ectopic expression of mutant p53^R248^ in p53-null SKOV-3 cells resulted in an increase of cell adhesion to mesothelial cells

**Figure 2:**
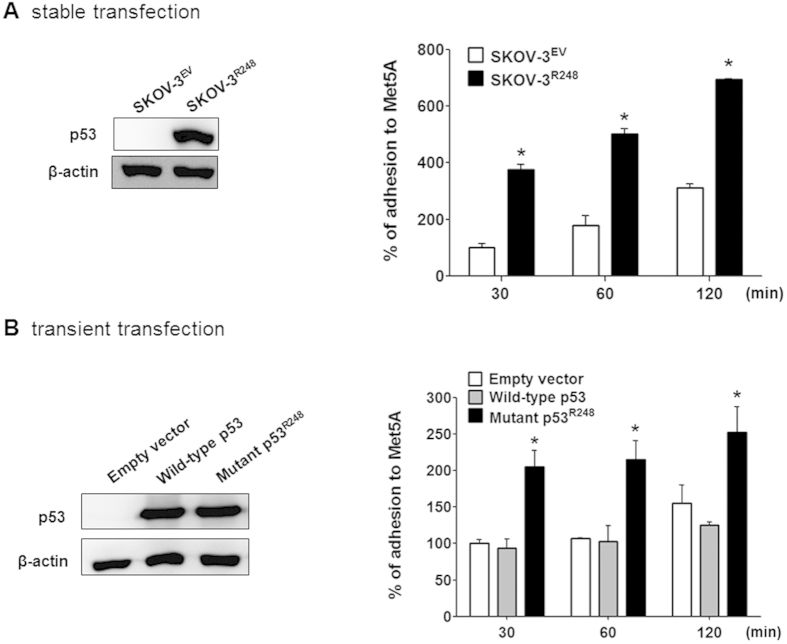
Effect of ectopic expression of mutant p53^R248^ in p53-null SKOV-3 cells on cell adhesion to mesothelial Met5A cells. (**A)** Levels of p53 were analysed by Western blotting using specific antibodies. Attachment assays were performed to investigate the adhesive ability of stably transfected SKOV-3^EV^ and SKOV-3^R248^ cells. (**B**) Levels of p53 were analysed by Western blotting using specific antibodies. Attachment assays were performed to investigate the adhesive ability of transiently transfected SKOV-3^EV^, SKOV-3^WT^, and SKOV-3^R248^ cells. Cells were cultured on Met5A cell layers in 96-well plates for the indicated time (30, 60, and 120 min). *P < 0.05 compared with the SKOV-3^EV^ group.

**Figure 3:**
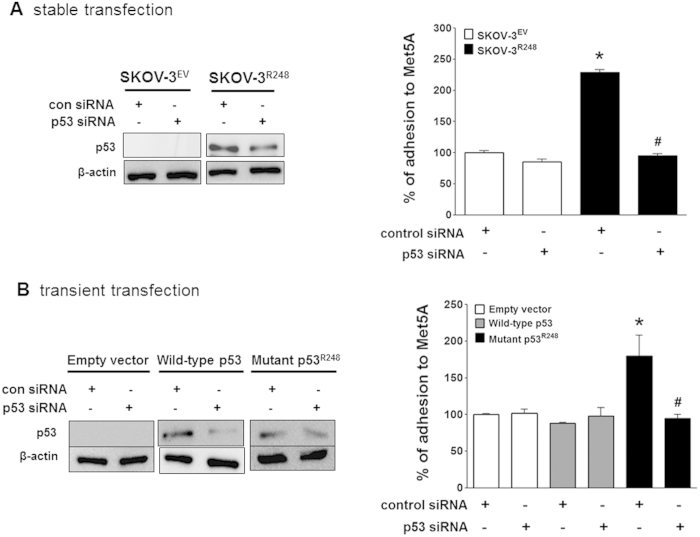
Effect of p53^R248^ down-regulation on mutant p53^R248^-induced ovarian cancer-mesothelial cell adhesion. Attachment assays were performed after transfection with p53 siRNA or control siRNA in stably. (**A**) or transiently (**B**) transfected SKOV-3 cells. Cells were cultured on Met5A cell layers in 96-well plates for 60 min. Levels of p53 were analysed after p53 siRNA transfection by Western blotting using specific antibodies. *P < 0.05 compared with the control siRNA-transfected SKOV-3^EV^ group and ^#^P < 0.05 compared with control siRNA-transfected SKOV-3^R248^ group.

Next, we investigated whether ectopic expression of mutant p53 affects cancer cell adhesion to mesothelial cells. First, SKOV-3^R248^ and SKOV-3^EV^ cells were established by stable transfection of p53-null SKOV-3 cells with the p53^R248^ mutant or control vector (empty vector), respectively. As observed in Fig. 2A, the stably transfected SKOV-3^R248^ cells were more adhesive than SKOV-3^EV^ cells. Similarly, transient transfection of SKOV-3 cells with mutant p53^R248^ resulted in a significant increase of cell adhesion to Met5A cells (Fig. 2B). These data suggest that ectopic expression of the mutant p53 increases ovarian cancer-mesothelial cell adhesion. To confirm the role of mutant p53^R248^ in ovarian cancer cell adhesion, cell adhesion was investigated after a knockdown of the mutant p53 using p53 siRNA. The p53^R248^-mediated cancer cell attachment to Met5A cells was completely inhibited by down-regulation of the p53 mutant (Fig. 3A,B). In contrast, transfection of SKOV-3^EV^ and SKOV-3^WT^ cells with p53 siRNA failed to show any significant change in cell adhesion.

### Integrin β4 is involved in p53^R248^-induced ovarian cancer-mesothelial cell adhesion

**Figure 4:**
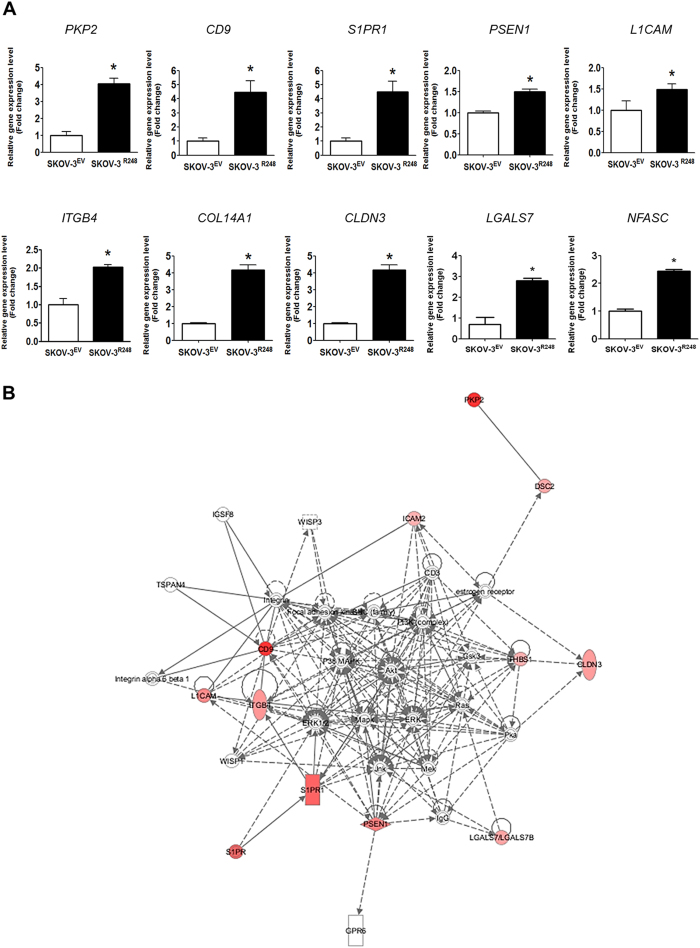
Ingenuity pathways analysis of adhesion-associated genes regulated by mutant p53^R248^. (**A**) Real-time RT-PCR shows the pattern of gene expression of ten selected genes, including *PKP2, CD9, S1PR1, PSEN1, L1CAM, ITGB4, COL14A1, CLDN3, LGALS7, and NFASC* in SKOV-3^R248^ and SKOV-3^EV^ cells. *P < 0.05 compared with the SKOV-3^EV^ group. (**B**) The genes involved in cell adhesion with an increase of at least 1.5-fold were analysed through the use of QIAGEN’s Ingenuity Pathways Analysis (IPA, QIAGEN Redwood City, www.qiagen.com/ingenuity). IPA system facilitates the identification of functional connections among the mutant p53^R248^-regulated genes with known relevance to cell adhesion. Only the top network, which had a score of 35, is shown. Red shapes, up-regulated genes in SKOV-3^R248^ cells; white shapes, molecules added from the Ingenuity Knowledge Base.

To identify the mutant p53^R248^-regulated genes and pathways that are involved in cell adhesion, we performed global transcript profiling to compare gene expression in SKOV-3^EV^ and SKOV-3^R248^ cells using the Agilent Whole Human Genome Microarray. Compared with SKOV-3^EV^, SKOV-3^R248^ cells showed up-regulation and down-regulation in 2737 genes with at least a 1.5-fold increase or decrease (data not shown). By applying DAVID analysis, several ontology groups showing a significant change after ectopic expression of mutant p53^R248^ were identified and ordered by *p* value for class enrichment significance in Suppl. Table S2. The top 10 ontology groups included Cell Adhesion and Biological Adhesion (Suppl. Table S2), and the same 146 adhesion-related genes fit into both classes (Suppl. Table S3). Among the 146 genes, ten up-regulated genes (*PKP2, CD9, S1PR1, PSEN1, L1CAM, ITGB4, COL14A1, CLDN3, LGALS7, and NFASC*) were selected for validation by quantitative real-time PCR. As shown in Fig. 4A, all ten genes showed statistically significant up-regulation in SKOV-3 cells after mutant p53^R248^ overexpression. These observations suggest that the mutant p53^R248^ regulated the expression of a number of adhesion-related genes that may play key roles in the mutant-mediated ovarian cancer-mesothelial cell adhesion. In addition, to identify the hidden connections among the regulated genes, we performed a network analysis of the up-regulated adhesion-related genes using the Ingenuity Pathways Analysis Systems (Fig. 4B). Several molecular hubs, including ERK1/2, p38, PKA, PI3K/Akt, and FAK, on which many pathways converge were identified in the network.

**Figure 5:**
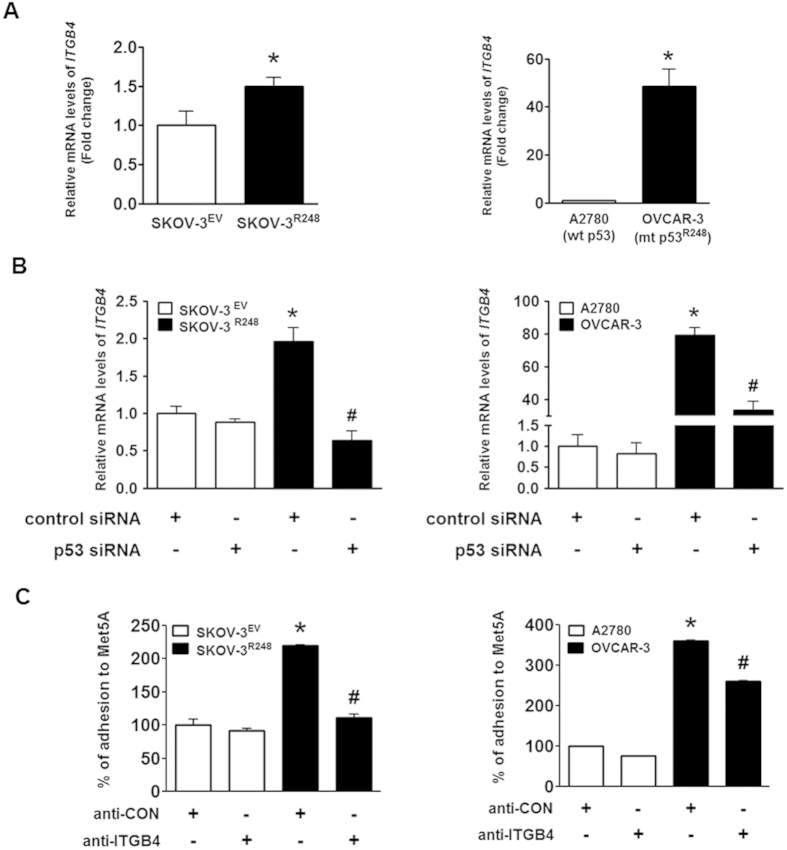
Involvement of integrin β4 in p53^R248^-induced ovarian cancer-mesothelial cell adhesion. (**A**) Real-time RT-PCR was performed to measure the mRNA levels of integrin β4 in transiently transfected SKOV-3 (SKOV-3^EV^ and SKOV-3^R248^), A2780, and OVCAR-3 cells. (**B**) The p53 siRNA was transiently transfected to suppress the levels of p53 in ovarian cancer cells. Real-time RT-PCR was performed to measure the mRNA levels of integrin β4 in SKOV-3^EV^, SKOV-3^R248^, A2780, and OVCAR-3 cells. (**C**) Cells were incubated with either integrin β4 blocking antibody anti-ITGB4 or control antibody (concentration-matched IgG control) for 30 min before and during the adhesion assay. *P < 0.05 compared with the control siRNA-transfected SKOV-3^EV^ and A2780 group; ^#^P < 0.05 compared with control siRNA-transfected SKOV-3^R248^ and OVCAR-3 group.

Integrins have been known to play an essential role in cancer cell adhesion^14^. In ovarian cancer, beta 4 integrin (integrin β4 encoded by *ITGB4*) was found to be overexpressed and to correlate with tumour aggressiveness^15^,^16^. In the present study, we demonstrated that integrin β4 is significantly up-regulated by mutant p53^R248^ in ovarian cancer cells (Fig. 4A and Suppl. Table S3). This observation is consistent with other findings that integrin β4 is involved in cell adhesion by regulating the cell-cell interaction in breast cancer^17^, colon cancer^18^, and pancreatic cancer^19^. In addition, the PI3K/Akt and FAK pathways that have been implicated in integrin-induced signal transduction have been identified as molecular hubs in the network of the mutant p53^R248^-regulated genes (Fig. 4B). More interestingly, a potential inhibitory effect of wild-type p53 on integrin β4 expression in cancer cells has been suggested^20^. These observations led us to hypothesise that integrin β4 may play a key role in the mutant p53^R248^-mediated ovarian cancer cell adhesion to mesothelial cells. Like stable expression (Fig. 4A and Suppl. Table S3), transient ectopic expression of p53^R248^ significantly increased integrin β4 expression (Fig. 5A). In addition, OVCAR-3 cells that endogenously overexpress p53^R248^ were found to express higher levels of integrin β4 than A2780 cells that express wild-type p53. Knockdown of the mutant using p53 siRNA significantly suppressed the levels of integrin β4 in SKOV-3^R248^ and OVCAR-3 cells (Fig. 5B). These data suggest that integrin β4 is regulated by p53^R248^ in ovarian cancer cells. Next, we investigated the effect of specific integrin β4 blockage antibody (anti-ITGB4) on p53^R248^-mediated ovarian cancer-mesothelial cell adhesion. Anti-ITGB4 was found to significantly inhibit adhesion of SKOV-3^R248^ and OVCAR-3 cells to Met5A cells, suggesting a role of integrin β4 in mutant p53-mediated ovarian cancer-mesothelial cell adhesion (Fig. 5C).

### The PI3K/Akt pathway is involved in p53^R248^-induced ovarian cancer-mesothelial cell adhesion

**Figure 6:**
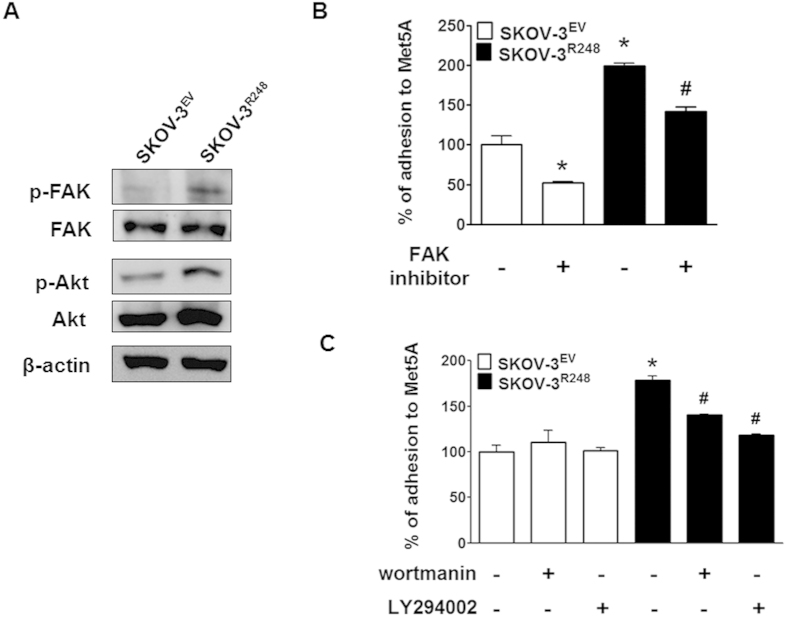
Involvement of Akt signalling in p53^R248^-induced ovarian cancer-mesothelial cell adhesion. (**A)** Western blot assays were performed to measure the phosphorylation of FAK and Akt in SKOV-3^EV^ and SKOV-3^R248^ cells. (**B)** After pretreatment with FAK inhibitor 14 (10 μM), wortmanin (1 μM), and LY294002 (5 μM) inhibitors, adhesion assays were performed using SKOV-3^R248^ and SKOV-3^EV^ cells. *P < 0.05 compared with the DMSO-treated SKOV-3^EV^ and A2780 group; ^#^P < 0.05 compared with the DMSO-treated SKOV-3^R248^ and OVCAR-3 group.

FAK, a downstream signalling molecule of integrin^21^, is known to play a key role in cell adhesion by phosphorylating downstream targets, such as PI3K/Akt^22^. Additionally, in this study, the ingenuity pathway analysis recognised FAK and Akt as molecular hubs for p53^R248^-regulated genes (Fig. 4B). We therefore investigated whether FAK and PI3K/Akt are involved in p53^R248^-mediated cell adhesion. In fact, ectopic expression of p53^R248^ induced a significant increase in the phosphorylated forms of both FAK and PI3K/Akt (Fig. 6A). Moreover, the PI3K/Akt inhibitors wortmannin and LY294002 significantly suppressed p53^R248^-induced cell adhesion to Met5A cells (Fig. 6B). However, the effect of the FAK inhibitor on SKOV-3^R248^ cell adhesion was comparable to that on SKOV-3^EV^ cell adhesion. These data suggest that PI3K/Akt, but not FAK, is a key molecule for p53^R248^-induced ovarian cancer-mesothelial cell adhesion.

## Discussion

Ovarian cancer is one of the most aggressive gynaecological malignancies, and its high frequency of mortality is most often a direct result of delays in diagnosis. Only 25% of ovarian cancers are diagnosed while the malignancy is still confined to the ovary, and the cure rate in these patients can reach 90%. The remaining 75% of ovarian tumours (stages III and IV) have spread beyond the ovary by the time of diagnosis, and the cure rate for these patients is less than 20%^23^. In contrast to that of most solid tumours, the metastatic spread of ovarian cancer rarely occurs through the vasculature; ovarian cancer primarily metastasises into the abdominal cavity. Stage III is characterised by peritoneal metastases throughout the abdominal cavity, and stage IV is defined by distant metastases. Thus, the metastatic spread of ovarian cancer is characterised by ascites and widespread peritoneal implantation, and successful adhesion is a key step in the metastasis of ovarian cancer cells. This unique metastatic mechanism seems to be associated with the poor chemotherapy response rate and patient prognosis in ovarian cancer^24^. In this regard, understanding the molecular mechanisms underlying this process may provide new therapeutic targets.

The concept that mutant p53 proteins gain tumour-promoting functions was established more than two decades ago by showing that mutant p53 has oncogenic effects. Many oncogenic functions of mutant p53 have been characterised in cell culture models, including an ability to promote scattering^25^, angiogenesis^26^, survival^27^, proliferation^28^, and tissue remodelling^29^. Enhanced chemoresistance^7^, mitogenic defects, and genomic instability^30^ have also been reported. Remarkably, increased tumour aggressiveness and higher metastatic potential are also hallmarks of p53 gain-of-function mutants. In fact, accumulating evidence suggested that mutant p53 can augment cell migration and invasion^31^,^32^. For example, Dong *et al*. reported that over-expression of p53 R175H significantly promoted cell migration and invasion and induced activation of the EGFR/PI3K/Akt pathway in endometrial cancer cells^33^. The expression of p53 R175H, R273H, or D281G in H1299 enhanced cell migration^34^, whereas the mutant p53 R248Q enhanced the invasiveness of lung cancer cells^35^. However, the mesothelial adhesion of cancer cells has been poorly studied, and it has not been determined whether mutant p53 plays a role in cell adhesion. A recent study reported an effect of a mutant p53 on the adhesion of H1299 lung cancer cells to the extracellular matrix fibronectin^36^. In that study, H1299 cells expressing mutant p53 (R175H, R273H, or D281G) showed enhanced adhesion to fibronectin-coated plates compared to control-transfected cells, but the exact mechanism of the response to mutant p53 was not elucidated. In the current study, ovarian cancer cells with abundant mutant p53 (OVCAR-3) were extremely adhesive to mesothelial cells when it compared to ovarian cancer cells with wild-type p53 (A2780) (Fig. 1C). In addition, the adhesion of cancer cells overexpressing mutant p53 (Hec1A, HT-29, and OVCAR-3 cells) was dramatically inhibited by down-regulation of the p53 mutant (Fig. 1C and Suppl. Fig. S1). Thus, we investigated whether the p53 mutation at codon 248 (denoted p53^R248^) plays a role in ovarian cancer adhesion to the mesothelium. Mutations in codon 248 in *TP53* are the common in human cancer and promote a gain of function in several cancers including ovarian cancer^37^. In fact, the p53^R248^ gain-of-function mutation has been reported to have various biological activities, including invasion^35^, tumour vascularisation^38^, up-regulation of chemokines^34^, and genetic instability^39^. Through our work, we demonstrated that mutant p53^R248^ expression was associated with increased mesothelial adhesion of ovarian cancer cells while transient transfection of SKOV-3 cells with wild-type p53 did not affect cancer cell adhesion (Figs 2B and 3B). We failed to establish stable SKOV-3 cell line with wild-type p53 due to growth retardation for clones with excess wild-type p53, which is well known to induce growth arrest and apoptosis.

The large percentage (~90%) of the p53 mutations found in human cancers map to the DNA-binding domain of p53 and ~40% of them occur at R248, R273, R175, R249, R282, and G245^40^. In human ovarian cancer, mutations in codon R248 are the second most common *TP53* alterations, with the most frequent mutations resulting in an amino acid change to tryptophan or glutamine (R248W or R248Q). In this study, we found that R248Q and R248W mutants promote cell adhesion to mesothelial cells in OVCAR-3 cells and SKOV-3 cells, respectively. The involvement of R248Q in cancer cell adhesion was also observed in endometrial cancer Hec1A cells (Suppl. Fig. S1). These data suggest that the nature of the substitution (tryptophan or glutamine) may not influence the adhesion-inducing activity of the resulting R248 mutant protein. Accumulating evidence suggests that different p53 mutants have a distinct activity profile while the p53 mutants have been believed to be equivalent. Considering that the codon 273 is the most commonly altered amino acid in ovarian cancer, we also investigated whether R273 mutant plays a role in ovarian cancer adhesion to the mesothelium. It was found that ectopic expression of R273H mutant in SKOV-3 cells significantly increased adhesion to Met5A cells (Suppl. Fig. S2). In addition, R273H seems to be associated with cell adhesion in colon cancer HT-29 cells (Suppl. Fig. S1). It is of note that a mutation at R175, the third most common *TP53* alterations in human ovarian cancer, did not show any significant change in ovarian cancer adhesion (data not shown). This observation may partly be explained by the fact that R175 mutant is classified differently from R248 and R273 mutants according to its structure. R175 is a conformational mutant while R248 and R273 are classified as DNA-contact mutant^41^. The underlying mechanism of the difference in oncogenic activities between the two types of mutants remains to be determined.

Since the mutant p53 gain-of-function properties were demonstrated, several underlying mechanisms have been proposed. A pivotal gain-of-function mechanism is the ability of the p53 mutants to regulate gene expression patterns. Numerous genes have been reported to be regulated by mutant p53, and many of those genes are associated in various ways with various stages of tumour progression, including proliferation, migration, invasion, and angiogenesis. For example, mutant p53 increases the expression of many proliferative and anti-apoptotic genes, including *CCNA, CCNB2, CDK1, CDC25C, PCNA, MYC, IGF1R, EGR1, ATF3, FAS, BCL2L, NFkB2,* and *ABCB1*^42^. In addition, *CXCL1, IL1B, IL6, IL8, SISP2,* and *VEGFA,* which are associated with invasiveness, inflammation, and angiogenesis in cancer, have been reported to be regulated by mutant p53. Considering that mutant p53 interacts with various transcription factors in a signal-dependent manner, the subset of genes that can be modulated by mutant p53 is likely to vary greatly among different cell types and cell contexts^42^. By comparing the gene profiles of p53 null SKOV-3 cells (transfected with only empty vector) and p53^R248^ overexpressing SKOV-3 cells, we found that mutant p53^R248^ expression was associated with changes in the expression of several genes. As expected, the gene ontology term analysis showed that a large number of the modulated genes are involved in adhesion (i.e., *PKP2, CD9, S1PR1, PSEN1, L1CAM, ITGB4, COL14A1, and CLDN3*). Among them, up-regulation of *IGTB4* is of interest because various integrins, including the gene product integrin β4, were previously reported to be involved in cancer cell adhesion^14^,^43^. In addition, integrins have been reported to be an important mediator of mesothelial adhesion of ovarian cancer cells^44^,^45^,^46^. For example, up-regulation of the fibronectin receptor, integrin α5β1, promotes the adhesion of ovarian cancer cells to secondary metastasis sites, including the omentum and peritoneum^44^,^45^. Integrin α4β1 and its adhesion receptor, cell adhesion molecule-1 (VCAM-1), were also reported to play a role in the interaction between cancer cells and the mesothelial cells^46^. Interestingly, it has been reported that integrin β4 is over-expressed in serous ovarian carcinoma^15^. Furthermore, a study has suggested that integrin β4 is transcriptionally repressed in tumours by wild-type p53 and HIPK2 to impair integrin β4-dependent tumour progression^20^. In that study, several colon cancer cell lines expressing mutant p53 (WiDr, HT-29, SW480, and GEO) showed high levels of integrin β4 expression, whereas wt p53-carrying cells (MIP, DLD-1, LoVo, and LNCaP) showed low levels of integrin β4 expression. However, the mechanism by which integrin β4 is up-regulated in ovarian cancer has never been investigated. In the present study, for the first time, we demonstrated that integrin β4 is up-regulated by mutant p53 expression and is involved in peritoneal adhesion of ovarian cancer cells. The anti-ITGB4 dramatically inhibited adhesion of SKOV-3^R248^ cells to Met5A cells. It is noteworthy that anti-ITGB4 treatment showed only partial inhibition of the cell adhesion in OVCAR-3 cells while knockdown of the mutant using p53 siRNA significantly suppressed the levels of integrin β4 in OVCAR-3 cells (Suppl. Fig. S3). One possible explanation would be that other adhesion molecules, such as cadherin, as well as integrin may play a role in p53 mutant-enhanced cell adhesion in OVCAR-3 cells. Recent studies suggested that SKOV-3 cells are possibly of clear cell or endometrioid origin while OVCAR-3 cells are derived from high-grade serous, the most common and deadliest subtype of epithelial ovarian cancer^47^,^48^. In this regard, the dissimilar molecular mechanism of action of the R248 mutants may reflect variances in the expression of targets of mutant p53 such as Tap63 and Tap73 in different subtypes of ovarian cancer. Further experimentation is required to confirm this hypothesis.

The most well established mechanism for the transcriptional effects of mutant p53 is associated with the ability of p53 mutants to interact with the p53 family members p63 and p73 and to alter their transcriptional activity^5^,^42^. Interestingly, the R248W p53 mutant has been shown to inhibit transactivation of p73beta, p73alpha, and p63, although it lacks sequence-specific DNA binding ability^49^,^50^. In addition, a study suggested that with the loss of wild-type p53 function, integrin β4 transcription is strongly activated by Tap63 and Tap73. These observations strongly support the possibility that mutant p53 may physically interact with p63/p73 or other transcription factors to regulate integrin β4 expression in ovarian cancer cells. The exact underlying molecular mechanism remains to be further investigated. The next question was how integrin β4 contributes to cell adhesion. Using interaction network analysis, several major molecular hubs were identified in this network, and they include PKA, FAK, MAPK, and PI3K/Akt. Among them, PI3K/Akt and FAK have been well documented to play a role in integrin-mediated cell adhesion^51^,^52^. For example, PI3K/Akt signalling was required for integrin-dependent attachment of OCUM-2MD3 gastric cancer cells^52^. Beviglia *et al*. reported that hepatocyte growth factor (HGF) induced integrin-mediated adhesion via FAK activation in MTKn3 breast cancer cells^51^. In the present study, we found that Akt signalling, but not FAK, is involved in p53^R248^-mediated ovarian cancer-mesothelial adhesion. In addition, anti-ITGB4 inhibited p53^R248^-induced Akt activation (Suppl. Fig. S4), suggesting that PI3K/Akt activation induced by the mutant may be mediated via integrin β4 expression. Akt-dependent, but FAK-independent, integrin-mediated adhesion has been reported^52^. Matsuoka *et al*. suggested that the PI3K/Akt signalling required for cancer cell adhesion was associated with integrin signalling through Src and vinculin but not FAK. It remains to be further demonstrated whether Src and/or vinculin mediate PI3K/Akt activation by integrin β4 in the mesothelial adhesion of ovarian cancer cells.

In summary, we have provided new evidence that the p53^R248^ gain-of-function mutant induced the adhesion of mesothelial cells in ovarian cancer. The p53^R248^ mutant, which activates intracellular integrin signalling, may provide a metastatic advantage to ovarian cancer cells in the peritoneal cavity. The above results demonstrate that specific inhibition of the mutant p53^R248^ or β4-integrin signalling may provide therapeutic strategies for ovarian cancer.

## Materials and Methods

### Materials

RPMI 1640 medium, foetal bovine serum (FBS), penicillin, and streptomycin were obtained from Life Technologies Inc. (Grand Island, NY, USA). Dimethyl sulfoxide (DMSO), RNase A, leupeptin, aprotinin, phenylmethylsulfonylfluoride, selective FAK inhibitor 14 (1,2,4,5-benzenetetramine tetrahydrochloride), and Triton X-100 were purchased from Sigma–Aldrich Co. (St Louis, MO, USA). The pCMV-Neo-Bam p53 R248W, pCMV-Neo-Bam p53 R273H, and pCMV-Neo-Bam p53 wild-type were obtained from Addgene (Cambridge, MA). CellTracker^TM^ was obtained from Invitrogen (Grand Island, NY, USA). The antibodies anti-p53 (DO-1, sc-126), anti-Akt, anti-focal adhesion kinase (FAK), integrin β4, and anti-β-actin were purchased from Santa Cruz Biotechnology (Santa Cruz, CA, USA). The antibodies anti-phospho-Akt and anti-phospho-FAK were purchased from Cell Signalling Technology (Beverly, MA, USA). Function-blocking antibody against the human integrin β4 was obtained from EMD Millipore (Billerica, MA, USA)^53^. The PI3K/Akt inhibitor LY294002 and wortmannin were obtained from Calbiochem (San Diego, CA, USA).

### Cell culture and transfection

The human ovarian cancer cell lines SKOV-3, A2780, and OVCAR-3, the human breast cancer cell line MCF-7, the human endometrial cancer cell line Hec1A, the human colon cancer cell line HT-29, and the human mesothelial cell line Met5A are originally from the American Type Culture Collection. The cells were cultured in RPMI 1640 supplemented with 5% FBS, penicillin (100 U/mL) and streptomycin sulfate (100 μg/mL). The cells were transfected with plasmid vector at a final concentration of 100 nM using polyethylenimine (Sigma–Aldrich Co.). Cells were plated in 6-well culture dishes and allowed to attach and grow for 24 h before transfection. Each transfection mixture was prepared by mixing the DNA (1 μg) and polyethylenimine (3 μl) in serum-free Opti-modified Eagle’s medium (Invitrogen) and incubated for 15 min at room temperature. The transfection mixture was slowly added to the cells, which were allowed to recover for an additional 24 h before experimental treatments. Stable clones (SKOV-3^EV^ and SKOV-3^R248^) were selected in the presence of 1 μg/ml neomycin and expanded, and gene expression was confirmed by Western blotting.

### Adhesion assay

The mesothelial Met5A cells were grown to confluence on 96-well plates. Ovarian cancer cells were detached by trypsinisation, washed with phosphate-buffered saline (PBS), and probed with 10 mM CellTracker^TM^ for 45 min at 37 °C. CellTracker^TM^-labelled cells were washed with RPMI 1640 medium containing 0.1% FBS to remove the free dye and added (2 × 10^4^ cells/well) to the mesothelial cells. After incubation at 37 °C for the indicated time, the non-adherent cells were removed by gentle washing, the fluorescence in each well was imaged, and the fluorescence was quantified in pixels using Scion Image Software (Scion Corp, Frederick, MD, USA). In the inhibition studies, Met5A and/or ovarian cancer cells were incubated with appropriate antibodies or inhibitors for 30 min before and during the adhesion assay.

### Gene knockdown using small interfering RNA

Small interfering RNAs (siRNAs) for p53 and control siRNA were synthesised by Bioneer technology (South Korea). Cells were transfected with siRNA at a final concentration of 100 nM using lipofectamine (Invitrogen), according to the manufacturer’s suggested protocol. Briefly, cells were plated in 6-well culture dishes and allowed to attach and grow for 24 h before transfection. Each transfection mixture was prepared by mixing the siRNA and lipofectamine in serum-free Opti-MEM and incubating for 15 min at room temperature. The transfection mixture was slowly added to the cells, which were allowed to recover for an additional 24 h before experimental treatments.

### RNA isolation and real-time RT-PCR

Total RNA was prepared using the TRIzol reagent (Invitrogen), according to the manufacturer’s instructions. Total RNA (2.5 μg) was reverse transcribed into first-strand cDNA (Amersham Pharmacia Biotech, Oakville, Canada), following the manufacturer’s procedure. The synthesised cDNA was used as a template for polymerase chain reaction (PCR) amplification. Real-time PCR was performed using a Thermal Cycler Dice Real Time PCR System (Takara, Japan). The primers used for SYBR Green real-time RT-PCR are listed in Supplementary Table S1. A dissociation curve analysis of all mRNA showed a single peak. The mean cycle threshold (Ct) of the gene of interest was calculated from triplicate measurements and normalised to the mean Ct of a control gene, *GAPDH*.

### Western blot analysis

The cells were washed with ice-cold PBS and extracted in protein lysis buffer (Intron Biotechnology, South Korea). The protein concentration was determined by the Bradford assay. The cell lysates were mixed with an equal volume of 5× sodium dodecyl sulfate (SDS) sample buffer, boiled for 4 min and then separated by electrophoresis on 10–12% SDS-polyacrylamide gels (SDS-PAGE). After electrophoresis, the proteins were transferred to polyvinylidene difluoride (PVDF) membranes. Each membrane was immunoblotted using specific primary antibodies at 4 °C overnight. After washing, the signals were detected with horseradish peroxidase-conjugated secondary antibody for 1 h and visualised using an ECL chemiluminescent system (Amersham Pharmacia Biotech, Canada). Following three washes in Tris-buffered saline-T, immunopositive bands were visualised by enhanced chemiluminescence and quantitated using an Image Quant LAS-4000 (GE Healthcare Life Science, WI, USA).

### Microarray analysis

The Agilent Whole Human Genome Microarray (Agilent, Foster City, CA, USA) was used to analyse the gene expression of SKOV-3^EV^ and SKOV-3^R248^ cells. RNA was prepared from the cells using TRIzol (Invitrogen). Labelling, hybridisation, and scanning of the arrays were performed according to the manufacturer’s protocols at E-biogen Inc. (South Korea). To screen for transcripts differentially expressed in SKOV-3^EV^ versus SKOV-3^R248^ cells, transcripts with a fold change of >1.5 were considered to be significantly regulated. The “Database for Annotation, Visualisation and Integrated Discovery” (DAVID) program^54^ was used for gene annotation. The “Functional Annotation Tool” in the online version of DAVID (http://david.abcc.ncifcrf.gov/) was run using the default parameters and focusing on the categories Gene-Ontology-Biological Process. The genes involved in cell adhesion with an increase of at least 1.5-fold were entered into the Ingenuity Pathways Analysis System. These genes, called focus genes, were overlaid onto a global molecular network developed from information updated in the Ingenuity Pathways Knowledge Base. Networks of these focus genes were then algorithmically generated based on their connectivity.

### Statistical analysis

Statistical data are presented as the mean ± S.D. of three individual experiments performed in triplicate. Statistical analysis was carried out using Student’s t-test or a one-way ANOVA, and the level of significance was set at a *P* value of <0.05.

## Additional Information

**How to cite this article**: Lee, J.-G. *et al*. Mutant p53 promotes ovarian cancer cell adhesion to mesothelial cells via integrin β4 and Akt signals. *Sci. Rep*. **5**, 12642; doi: 10.1038/srep12642 (2015).

## Supplementary Material

Supplementary Information
