# Supplementary material for: Mutant p53 promotes ovarian cancer cell adhesion to mesothelial cells via integrin β4 and Akt signals
Source: Sci Rep. 2015 Jul 30;5:12642. doi: 10.1038/srep12642 (PMC4649895; doi:10.1038/srep12642)
Supplement: Supplementary Information [file srep12642-s1.pdf]

Mutant p53 promotes ovarian cancer cell adhesion to mesothelial cells via integrin β4 and Akt signals

Jong-Gyu Lee[1,2], Ji-Hye Ahn[1,2], Tae Jin Kim[3], Jae Ho Lee[4], and Jung-Hye Choi [1,2]

Table S1. Primer sequences for real-time PCR analysis

Gene	Sense primer	Antisense primer
PKP2	*GCAAATGGTTTGCTCGATTT*	*GGCTGGTAATCTGCAATGGT*
CD9	*TTCGGCCCAGGCTAAGTTAG*	*CGGCAAGCCAGAAGATGAAG*
S1PR1	*AAATTCCACCGACCCATGTA*	*AGTTATTGCTCCCGTTGTGG*
PSEN1	*ATCTAATGGACGACCCCAGG*	*CCCATCCTTCCGGGTATAAA*
L1CAM	*TCACGGGCAACAACAGCAACT*	*CGGCTTCCTGTCAATCATGCT*
ITGB4	*CATGAGGCCTGAGAAGCTGA*	*ATCCAGGTTGCCTGAGATCC*
COL14A1	*GGTGGAAAACCAGACGAGGT*	*CAGAAGCCATCGGTAAAGCA*
CLDN3	*CAACATCATCACGTCGCAGA*	*CTGAAGGTCCTGTGGCAGTG*
LGALS7	*TTCAACAGCAAGGAGCAAGG*	*GAAGTGGTGGTACTGGGCGT*
NFASC	*GGGCTGTGTACCAGTGCAAC*	*GGATGGGAGACCCAAAGAAA*
GAPDH	*GAGTCA ACGGATTTGGTCGT*	*TTGATTTTGGAGGGATCTCG*

Table S2. Analysis of gene ontology by microarray hybridisation of RNA from SKOV-3 cells expressing mutant $p53^{R248}$ compared with RNA from backbone vector transfected cells

Function	Related gene number	%	*p* value
Cell Adhesion	146	5.4	3.80E-10
Biological Adhesion	146	5.4	4.10E-09
Blood Vessel Development	64	2.4	5.10E-08
Vasculature Development	65	2.4	5.60E-08
Regulation of Cell Motion	54	2	5.80E-08
Extracellular Structure Organization	46	1.7	4.80E-07
Homophilic Cell Adhesion	38	1.4	2.70E-06
Blood Vessel Morphogenesis	53	2	2.90E-06
Regulation of Cell Proliferation	147	5.5	3.60E-06
Cell-Cell Adhesion	64	2.4	4.40E-06

The 2737 genes found to be up-regulated or down-regulated after ectopic expression of $p53^{R248}$ were functionally classified by DAVID [http://david.abcc.ncifcrf.gov/] into Biological Process classes. The table shows the number and percentage of the significantly modulated genes that fit into each ontology function class as well as the modified Fisher exact *p* value for the class enrichment significance.

Table S3. Summary of 146 adhesion-related genes determined by DAVID

No	Gene symbol	Gene description	List ID	Fold change
1	PKP2	plakophilin 2	NM_004572	3.87
2	CD9	CD9 molecule	NM_001769	3.84
3	S1PR1	sphingosine-1-phosphate receptor 1	NM_001400	2.92
4	PSEN1	presenilin 1	AJ008005	2.44
5	L1CAM	L1 cell adhesion molecule	NM_024003	2.25
6	ITGB4	integrin, beta 4	NM_000213	1.96
7	COL14A1	collagen, type XIV, alpha 1	NM_021110	1.93
8	CLDN3	claudin 3	NM_001306	1.87
9	LGALS7, LGALS7B	lectin, galactoside-binding, soluble, 7; lectin, galactoside-binding, soluble, 7B	NM_002307	1.75
10	NSASC	neurofascin homolog (chicken)	NM_001005388	1.74
11	DSC2	desmocollin 2	NM_024422	1.66
12	DPT	dermatopontin	NM_001937	1.63
13	ZAN	zonadhesin	NM_173059	1.61
14	PCDHGB2	protocadherin gamma subfamily B, 2	NM_032096	1.56
15	CNTNAP3, CNTNAP3B	contactin associated protein-like 3; contactin associated protein-like 3B	NM_033655	1.55
16	THBS1	thrombospondin 1	NM_003246	1.55
17	ICAM2	intercellular adhesion molecule 2	NM_000873	1.52
18	CYR61	cysteine-rich, angiogenic inducer, 61	NM_001554	1.52
19	PCDHA1, PCDHA4	protocadherin alpha 1; protocadherin alpha 4	NM_031410	0.67
20	ABL2	v-abl Abelson murine leukemia viral oncogene homolog 2 (arg, Abelson-related gene)	NM_001100108	0.67
21	LAMC2	laminin, gamma 2	NM_018891,<br>NM_005562	0.67,<br>0.57
22	PVRL3	poliovirus receptor-related 3	NM_015480	0.66
23	DSG2	desmoglein 2	NM_001943	0.66
24	BCL2L11	BCL2-like 11 (apoptosis facilitator)	NM_207002	0.66
25	SLURP1	secreted LY6/PLAUR domain containing 1	NM_020427	0.66
26	CASK	calcium/calmodulin-dependent serine protein kinase (MAGUK family)	NM_003688	0.66
27	PCDHGA8	protocadherin gamma subfamily A, 8	NM_032088	0.66
28	FERMT1	fermitin family homolog 1 (Drosophila)	NM_017671	0.65
29	PTPRM	protein tyrosine phosphatase, receptor type, M	NM_002845	0.65
30	ITGAE	integrin, alpha E (antigen CD103, human mucosal lymphocyte antigen 1; alpha polypeptide)	NM_002208	0.65
31	CNTN6	contactin 6	NM_014461	0.65
32	CD2AP	CD2-associated protein	NM_012120	0.65
33	HAPLN3	hyaluronan and proteoglycan link protein 3	NM_178232	0.65
34	ROCK1, ROCK1P1	similar to Rho-associated, coiled-coil containing protein kinase 1;<br>Rho-associated, coiled-coil containing protein kinase 1	NM_005406	0.65
35	PCDHA3	protocadherin alpha 3	NM_031497	0.65
36	LAMB2	laminin, beta 2 (laminin S)	NM_002292	0.65

Table S3. (continued)

No	Gene symbol	Gene description	List ID	Fold change
37	ATP2C1	ATPase, Ca++ transporting, type 2C, member 1	NM_001001485	0.65
38	ITGB3	integrin, beta 3 (platelet glycoprotein IIIa, antigen CD61)	NM_000212	0.64
39	F8	coagulation factor VIII, procoagulant component	NM_000132	0.64
40	EMR1	egf-like module containing, mucin-like, hormone receptor-like 1	NM_001974	0.64
41	RET	ret proto-oncogene	NM_020630	0.64
42	SRPX	sushi-repeat-containing protein, X-linked	NM_007269	0.64
43	STXBP3	syntaxin binding protein 3	NM_007269	0.64
44	DLC1	deleted in liver cancer 1	NM_182643	0.64
45	SGCE	sarcoglycan, epsilon	NM_001099401	0.64
46	TGFBI	transforming growth factor, beta-induced, 68kDa	NM_000358	0.64
47	MPZL3	myelin protein zero-like 3	AK095399	0.64
48	ADMA23	ADAM metallopeptidase domain 23	NM_003812	0.63
49	FBLIM1	filamin binding LIM protein 1	NM_017556	0.63
50	EGFR	epidermal growth factor receptor (erythroblastic leukemia viral (v-erb-b) oncogene homolog, avian)	NM_201283	0.63
51	PCDHGA7	protocadherin gamma subfamily A, 7	NM_032087	0.63
52	RPSA, RPSAP12, RPSAP15, RPSAP18, RPSAP19, RPSA P29, RPSAP58, RPSAP61, RPSAP8, RPSAP9	ribosomal protein SA pseudogene 9; ribosomal protein SA pseudogene 8; ribosomal protein SA pseu dogene 58; ribosomal protein SA pseudogene 19; ribosomal protein SA pseudogene 18; ribosomal pr otein SA; ribosomal protein SA pseudogene 15; ribosomal protein SA pseudogene 61; ribosomal prot ein SA pseudogene 29; ribosomal protein SA pseudogene 12	NM_002295	0.63
53	ABL1	c-abl oncogene 1, receptor tyrosine kinase	NM_005157	0.63
54	ITGA11	integrin, alpha 11	NM_001004439	0.63
55	TAOK2	TAO kinase 2	NM_004783	0.62
56	PTPRK	protein tyrosine phosphatase, receptor type, K	NM_002844	0.62
57	BMP1	bone morphogenetic protein 1	NM_001199	0.62
58	CXCL12	chemokine (C-X-C motif) ligand 12 (stromal cell-derived factor 1)	NM_001033886	0.62
59	ANTXR1	anthrax toxin receptor 1	NM_032208	0.62
60	PKD2	polycystic kidney disease 2 (autosomal dominant)	NM_000297	0.62
61	ADAM15	ADAM metallopeptidase domain 15	NM_207191	0.62
62	COL27A1	collagen, type XXVII, alpha 1	AK021957	0.62
63	FCGBP, LOC100133944	Fc fragment of IgG binding protein; similar to IgGFc-binding protein precursor (FcgammaBP) (Fcgam ma-binding protein antigen)	NM_003890	0.62
64	ARHGAP5	Rho GTPase activating protein 5	NM_001030055	0.62
65	EPDR1	ependymin related protein 1 (zebrafish)	NM_017549	0.62
66	FNDC3A	fibronectin type III domain containing 3A	NM_001079673	0.61
67	MSN	moesin	NM_002444	0.61
68	CLDN14	claudin 14	NM_144492	0.61
69	PCDHGA2	protocadherin gamma subfamily A, 2	NM_032009	0.61
70	CLSTN1	calsyntenin 1	NM_001009566	0.61
71	CD99L2	CD99 molecule-like 2	NM_031462	0.61
72	LAMA3	laminin, alpha 3	NM_198129	0.60

Table S3. (continued)

No	Gene symbol	Gene description	List ID	Fold change
73	CHST4	carbohydrate (N-acetylglucosamine 6-O) sulfotransferase 4	NM_005769	0.60
74	DSCAML1	Down syndrome cell adhesion molecule like 1	NM_020693	0.60
75	BCL2	B-cell CLL/lymphoma 2	NM_000633	0.59
76	CDH2	cadherin 2, type 1, N-cadherin (neuronal)	NM_001792	0.59
77	PCDHB2	protocadherin beta 2	NM_018936	0.59
78	PCDH19	protocadherin 19	NM_020766	0.59
79	NID2	nidogen 2 (osteonidogen)	NM_007361	0.58
80	ADAM8	ADAM metallopeptidase domain 8	NM_001109662	0.58
81	RAPH1	Ras association (RalGDS/AF-6) and pleckstrin homology domains 1	NM_203365, NM_213589	0.58, 0.47
82	ITGB1	integrin, beta 1 (fibronectin receptor, beta polypeptide, antigen CD29 includes MDF2, MSK12)	NM_133376	0.58
83	AGGF1	angiogenic factor with G patch and FHA domains 1	NM_018046	0.58
84	PCDHB13	protocadherin beta 13	NM_018933	0.58
85	SCARB2	scavenger receptor class B, member 2	NM_005506	0.58
86	PCDHA7	protocadherin alpha 7	NM_031852	0.57
87	CLDN12	claudin 12	NM_012129	0.57
88	COL12A1	collagen, type XII, alpha 1	NM_004370	0.57
89	TNC	tenascin C	NM_002160	0.57
90	KITLG	KIT ligand	NM_000899	0.56
91	ALCAM, LOC100133690	hypothetical protein LOC100133690; activated leukocyte cell adhesion molecule	NM_001627	0.56
92	DCHS1	dachsous 1 (Drosophila)	NM_003737	0.56
93	CLDN1	claudin 1	NM_021101	0.56
94	COL7A1	collagen, type VII, alpha 1	NM_000094	0.56
95	LOXL2	lysyl oxidase-like 2	NM_002318	0.54
96	PTPRC	protein tyrosine phosphatase, receptor type, C	NM_002838	0.54
97	LPP	LIM domain containing preferred translocation partner in lipoma	NM_005578	0.54
98	LEF1	lymphoid enhancer-binding factor 1	NM_016269	0.53
99	FAT1	FAT tumor suppressor homolog 1 (Drosophila)	NM_005245	0.53
100	COL11A2	collagen, type XI, alpha 2	NM_080680	0.52
101	LAMC1	laminin, gamma 1 (formerly LAMB2)	NM_002293	0.52
102	ITGA10	integrin, alpha 10	NM_003637	0.52
103	CHST10	carbohydrate sulfotransferase 10	NM_004854	0.52
104	MYBPC3	myosin binding protein C, cardiac	NM_000256	0.51
105	LAMB1	laminin, beta 1	NM_002291	0.51
106	PCDHGA3	protocadherin gamma subfamily A, 3	NM_032011	0.51
107	CDH11	cadherin 11, type 2, OB-cadherin (osteoblast)	NM_001797	0.51
108	MPZL2	myelin protein zero-like 2	NM_005797	0.51
109	PCDH1	protocadherin 1	NM_002587	0.51
110	CLDN4	claudin 4	NM_001305	0.50

Table S3. (continued)

No	Gene symbol	Gene description	List ID	Fold change
111	EDIL3	EGF-like repeats and discoidin I-like domains 3	NM_005711	0.50
112	PCDHB11	protocadherin beta 11	NM_018931	0.50
113	PCDHB8	protocadherin beta 8	NM_019120	0.50
114	CDH6	cadherin 6, type 2, K-cadherin (fetal kidney)	NM_004932	0.49
115	PCDHB5	protocadherin beta 5	NM_015669	0.48
116	IGFBP7	insulin-like growth factor binding protein 7	NM_001553	0.48
117	PCDHB10; PCDHB9	protocadherin beta 10; protocadherin beta 9	NM_019119	0.48
118	F11R	F11 receptor	NM_016946	0.48
119	PCDH7	protocadherin 7	NM_002589	0.48
120	HSPG2	heparan sulfate proteoglycan 2	NM_005529	0.47
121	COL5A1	collagen, type V, alpha 1	NM_000093	0.47
122	SPP1	secreted phosphoprotein 1	NM_001040058	0.47
123	NRP1	neuropilin 1	NM_003873	0.47
124	CLDN7	claudin 7	NM_001307	0.46
125	NCAM2	neural cell adhesion molecule 2	NM_004540	0.46
126	DST	dystonin	NM_020388,<br>NM_015548	0.46,<br>0.54
127	WISP2	WNT1 inducible signaling pathway protein 2	NM_003881	0.46
128	VCAN	versican	NM_004385	0.45
129	B4GALT1	UDP-Gal:betaGlcNAc beta 1,4- galactosyltransferase, polypeptide 1	NM_001497	0.45
130	PCDHB16	protocadherin beta 16	NM_020957	0.44
131	LAMA4	laminin, alpha 4	NM_001105209	0.44
132	NRP2	neuropilin 2	NM_201264,<br>NM_201266	0.43,<br>0.45
133	NLGN1	neuroligin 1	NM_014932	0.42
134	PCDHB14	protocadherin beta 14	NM_018934	0.41
135	COL8A1	collagen, type VIII, alpha 1	NM_001850	0.41
136	IL32	interleukin 32	NM_001012633,<br>NM_001012631	0.40,<br>0.38
137	ITGAV	integrin, alpha V (vitronectin receptor, alpha polypeptide, antigen CD51)	NM_002210	0.39
138	COL16A1	collagen, type XVI, alpha 1	NM_001856	0.38
139	PDPN	podoplanin	NM_198389	0.37
140	PCDHB15	protocadherin beta 15	NM_018935	0.37
141	AMTN	amelotin	NM_212557	0.32
142	CADM1	cell adhesion molecule 1	NM_014333	0.31
143	FLRT3	fibronectin leucine rich transmembrane protein 3	NM_198391	0.28
144	SPOCK1	sparc/osteonectin, cwcv and kazal-like domains proteoglycan (testican) 1	NM_004598	0.27
145	CDH10	cadherin 10, type 2 (T2-cadherin)	NM_006727	0.25
146	PCDHB6	protocadherin beta 6	NM_018939	0.24

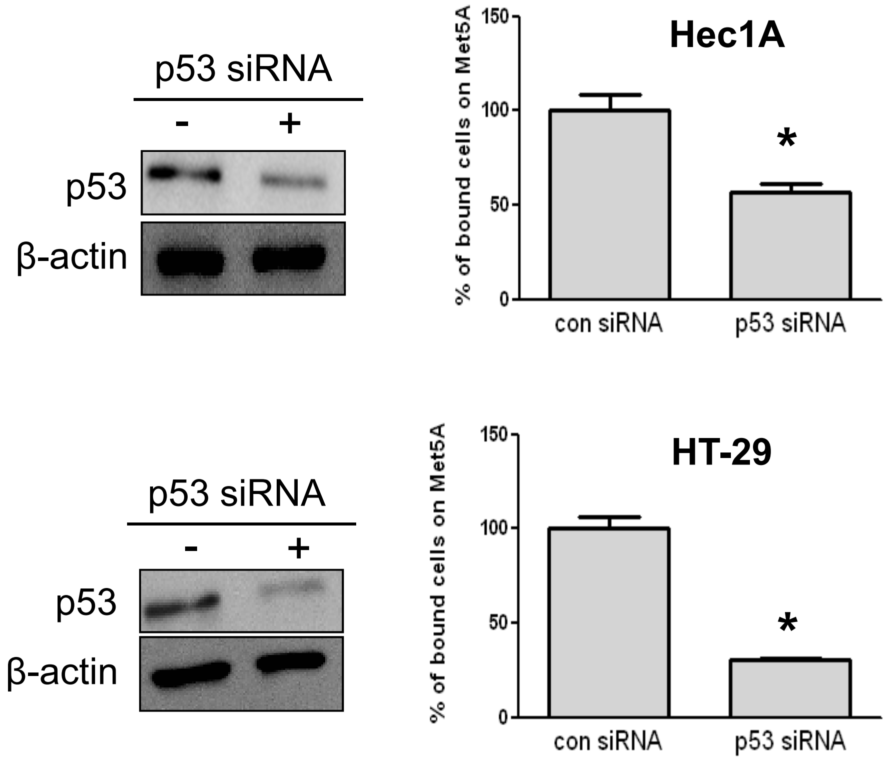

Figure. S1. Effect of knockdown of the mutant p53 on adhesion of endometrial cancer Hec1A (R248Q) and colon cancer HT-29 (R273H) cells

Attachment assays were performed after transfection with p53 siRNA or control siRNA in Hec1A and HT-29 cells. Cells were cultured on Met5A cell layers in 96-well plates for 60 min. Levels of p53 were analysed after p53 siRNA transfection by Western blotting using specific antibodies. *P < 0.05 compared with the control siRNA-transfected group.

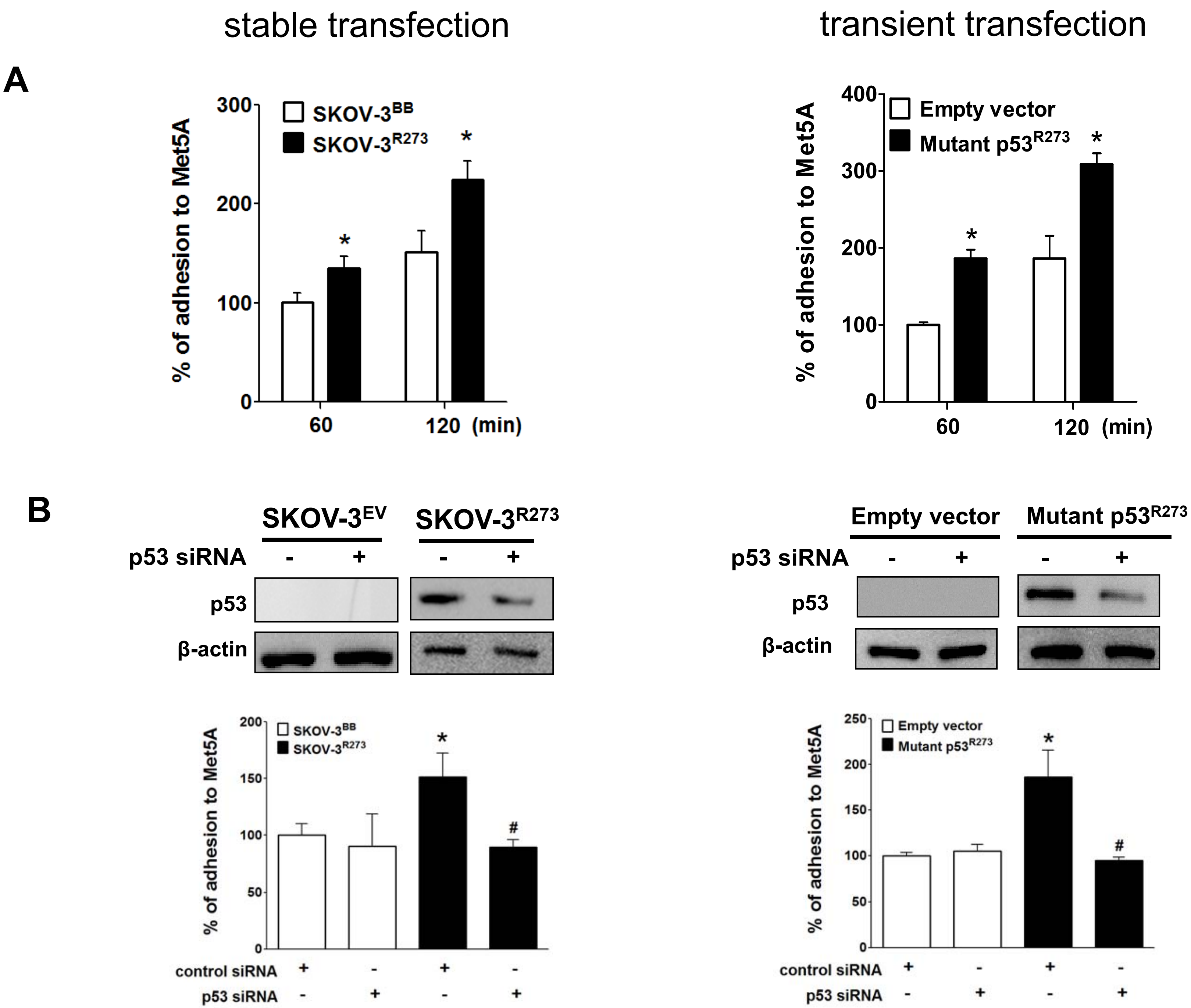
stable transfection
transient transfection
A
% of adhesion to Met5A
SKOV-3BB
SKOV-3R273
60
120 (min)
Empty vector
Mutant p53R273
B
SKOV-3EV
SKOV-3R273
p53 siRNA
p53
β-actin
Empty vector
Mutant p53R273
control siRNA
p53 siRNA

Figure. S2. Effect of ectopic expression of mutant p53^{R273} on cell adhesion to mesothelial Met5A cells

A. Attachment assays were performed to investigate the adhesive ability of stably or transiently transfected SKOV-3 cells. Cells were cultured on Met5A cell layers in 96-well plates for the indicated time (60 and 120 min). *P < 0.05 compared with the SKOV-3^{EV} group. **B.** Attachment assays were performed after transfection with p53 siRNA or control siRNA in stably or transiently transfected SKOV-3 cells. Cells were cultured on Met5A cell layers in 96-well plates for 60 min. Levels of p53 were analysed after p53 siRNA transfection by Western blotting using specific antibodies. *P < 0.05 compared with the control siRNA-transfected SKOV-3^{EV} group and $^{\#}$P < 0.05 compared with control siRNA-transfected SKOV-3^{R273} group.

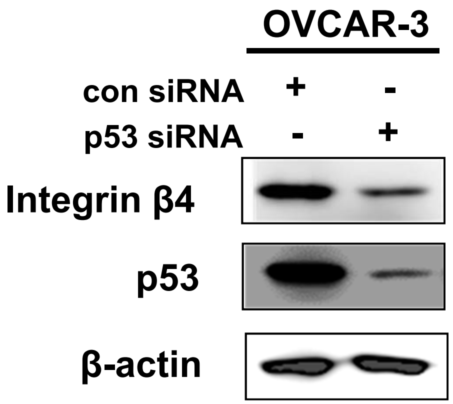

Figure S3. Effect of knockdown of the mutant p53 on the protein levels of integrin β4 and $p53^{R248}$ in OVCAR-3 cells. Levels of p53 and integrin β4 were analysed after p53 siRNA transfection by Western blotting using specific antibodies.

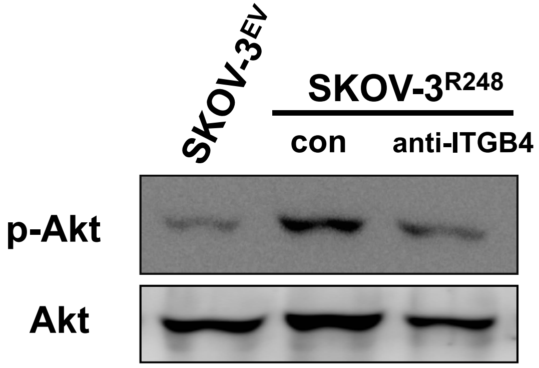

Figure S4. Effect of anti-ITGB4 on Akt activation in SKOV-3^{R248} cells

Cells were incubated with integrin β4 blocking antibody anti-ITGB4. Western blot assays were performed to measure the phosphorylation of FAK and Akt in SKOV-3^{EV} and SKOV-3^{R248} cells.
